# CT texture analysis of pediatric teratomas—associations with identification and grading of immature teratoma

**DOI:** 10.1186/s12880-025-01764-4

**Published:** 2025-07-01

**Authors:** Xinxin Qi, Xiaoyu Wang, Wen Zhao, Songyu Teng, Guanglun Zhou, Hongwu Zeng

**Affiliations:** 1 China Medical University, No.77 Puhe Road, Shenbei New District, Shenyang, 110122 China; 2 Department of Radiology, Shenzhen Children’s Hospital, 7019 Yitian Road, Futian District, Shenzhen, 518038 China; 3 Shantou University Medical College, Shantou University, 22 Xinling Road, Jinping District, Shantou, 515041 China; 4 Department of Urology and Laboratory of Pelvic Floor Muscle Function, Shenzhen Children’s Hospital, 7019 Yitian Road, Futian District, Shenzhen, 518038 China; 5 Radiology Department, Shenzhen Children’s Hospital, Affiliated to Shantou University Medical College, Guangdong, China

**Keywords:** Immature teratoma, CT texture analysis, Differential diagnosis, Tumor grading, Fat, Calcification, Solid component

## Abstract

**Background:**

Teratomas are categorized into mature teratomas (MT) and immature teratomas (IT) of grades I-III based on the content of immature tissues. The existing diagnostic methods are not comprehensive and objective enough. This study aims to utilize computed tomography texture analysis (CTTA) to exploring heterogeneity of tumor components and enhance the preoperative identification and grading of IT.

**Methods:**

Between 2019 and 2023, 52 patients with pathologically confirmed MT (*n* = 26) and IT (*n* = 26) underwent preoperative CT scans. Fat, calcification, and solid components of intratumoral components were extracted using 3D slicer. CT features including size and total volume, as well as 75 texture features were analyzed. Comparisons of these features were performed between the IT and MT groups and within the IT groups. Logistic regression models were constructed and the area under the curve (AUC) was used to evaluate the effectiveness of these models. Statistical significance was set at *p* < 0.05.

**Results:**

CT features showed that, IT group exhibited greater calcification size (*p* = 0.012), larger calcification volume (*p* = 0.003), and larger solid component volume (*p* < 0.001) than MT group. Texture features showed 22, 30, and 43 differential texture features for fat, calcification, and solid components between IT and MT group, respectively (*p* < 0.05). Among these, the neighborhood gray tone difference matrix busyness (NGTDM_busyness) feature for solid components was significantly higher in the IT group than in the MT group (*p* < 0.001) and higher in grade II than in grade I within the IT groups (*p* = 0.020). Logistic regression analysis indicated that IT identification efficacy of fat, calcifications, and solid components models were 0.778, 0.774, and 0.976, respectively.

**Conclusions:**

CTTA is an effective method for IT identification and grading, with NGTDM features holding unique value. Among tumor components, the solid components demonstrate excellent diagnostic value.

## Background

Teratoma is the most common germ cell tumor in children, composed of the inner, middle, and outer germ layers. Based on the presence and content of immature tissues within the tumor, teratomas are classified into mature teratoma (MT, grade 0) and immature teratoma (IT), with IT further categorized into grades I, II, and III [1]. According to data provided by the MaGIC (Malignant Germ Cell International Collaborative) in 2020 [2], MT has a relatively favorable prognosis, with a 5-year event-free survival (EFS) of 92.2%. In contrast, IT carries a higher risk of recurrence and metastasis, particularly grade III IT, which has a recurrence rate of up to 20%, leading to a decreased 5-year EFS of 85.9% and a relatively poorer prognosis [3]. Therefore, accurate preoperative identification and grading of IT plays a key role for predicting recurrence and guiding clinical management in pediatric patients.

Current diagnostic tools have significant limitations. Alpha-fetoprotein (AFP), commonly used in screening, lacks specificity and cannot grade IT. Postoperative pathology, the gold standard for classification, is prone to sampling bias due to tumor heterogeneity and subjective interpretation of histological features [1]. Recent imaging advances show promise. A 2023 multicenter study (*n* = 632) identified solid components, polymorphic calcification, and fat distribution in CT images as independent IT risk factors [4], but visual assessment remains subjective. These methodological gaps underscore the urgent need for an objective, comprehensive preoperative diagnostic approach.

Radiomics, introduced in 2012, offers a solution by converting visual imaging data into quantifiable features, enabling the characterization of tumor heterogeneity [5]. This method allows for an in-depth examination of the temporal and spatial heterogeneity of lesions and can differentiate malignant from benign tumors, predict preoperative pathological findings, and forecast treatment outcomes [6]. CT texture analysis (CTTA), a radiomics feature extraction technique, evaluates pixel grayscale intensity and distribution heterogeneity in conventional CT images [7]. A study published in “*Radiology*” demonstrated that CTTA could differentiate fat-poor renal angiomyolipoma from renal cell carcinoma with a discrimination efficacy of 0.89 [8].

Building upon this foundation, we hypothesize that CTTA may serve as a preoperative tool to characterize the heterogeneity of tumor components—including fat, calcification, and solid component—within intratumoral regions, thereby improving diagnostic accuracy. Our study aims to integrate CTTA to explore specific radiological biomarker of intratumoral components and construct a logistic regression model to assess their diagnostic efficacy. If validated, this approach could provide a noninvasive method to objectively and comprehensively assist in IT grading, predict recurrence risk, and refine prognostic stratification in pediatric populations, addressing current diagnostic limitations and advancing personalized therapeutic strategies.

## Methods

### Patient information

This was a retrospective case-control study approved by the institutional review board (IRB No. 2022021). Patients were identified by searching the pathology database from one academic tertiary care institution (****) between January 2019 and December 2023. The inclusion criteria were as follows: (1) Pathological confirmation of IT through surgical resection or biopsy; (2) Complete preoperative CT examination; (3) No chemotherapy before CT examination. The exclusion criteria were as follows: (1) IT pathological results not further graded according to the Norris grading system [9]; (2) Non-standard CT scans or images of insufficient quality. A total of 26 IT patients were ultimately included, with an additional 26 patients pathologically diagnosed with MT included as the control group.

### CT examination

CT scans were performed using a 64-slice spiral CT scanner (GE Optima, United States). The position of examination depended on the primary site, including the head, chest, abdominal and pelvic regions. Imaging parameters were set as follows: spiral scanning, tube voltage 120 kV, tube current 160-180 mA, pitch 1.375, matrix 512 × 512, scan layer thickness 5.0 mm, layer spacing 0.5 mm, and standard algorithm reconstruction with a reconstruction layer thickness of 1.25 mm.

### Radiology features extraction and model construction

Two senior radiologists observed, measured and delineated the teratoma lesions based on plain CT images with a layer thickness of 5.0 mm. In case of any disputes, a consensus diagnosis was reached through discussion.

#### CT features extraction

The measured parameters included the size and total volume of fat, calcification, and solid components. The size of fat and calcifications were represented by the maximum diameter on axial views. The total volume of fat, calcifications, and solid components was calculated using the 3D Slicer software (Version 4.11.0, https://www.slicer.org). As for tissue component, fat, calcification, and solid components were defined according to its CT values, setting as -144 to-20HU [10], ≥ 130HU [11], and 30–60 HU respectively. Hemorrhagic areas were excluded using contrast-enhanced images.

**Fig. 1:**
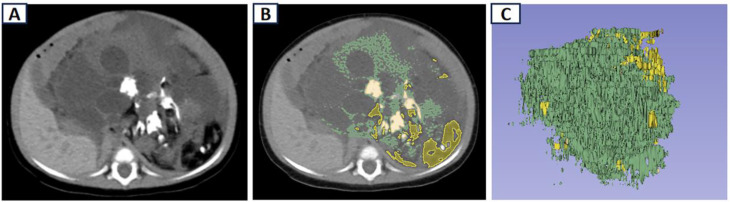
Delineation of teratoma VOI on CT. (**A**) CT; (**B**) semi-automatic delineation of VOIs of fat, calcification and solid component; (**C**) the VOI obtained. VOI, volume of interest; CT, Computed tomography; VOI, volume of interest

The volume of interest (VOI) of fat, calcification, and solid components was obtained semi-automatically based on the above range of CT values(Fig. 1), and the total volume of these components was calculated by the segment statistics module within the software.

#### Texture features extraction

These VOI images obtained through the 3D Slicer above were uploaded to the RIAT (Radiomics Intelligent Analysis Toolkit software) [12]. After resampling all images to 1 × 1 × 1 mm³, 75 texture features (https://pyradiomics.readthedocs.io/en/latest/) of fat, calcification, and solid components were extracted, including 24 Gy level co-occurrence matrix features (GLCM), 14 Gy level dependence matrix features (GLDM), 16 Gy level run length matrix features (GLRLM), 16 Gy level zone length matrix features (GLZLM), and 5 neighborhood gray tone difference (NGTDM) features.

#### Model construction and evaluation: (Fig. 2)

(I) First of all, features selection:

① Difference test: Mann-Whitney test was used for inter-group comparisons between MT and IT, and Kruskal-Wallis test was used for intra-group comparisons within different grade ITs. Image features with significant inter-group and intra-group differences (*p* < 0.05) were retained.

② Collinearity analysis: Collinearity analysis was performed on the retained features, the features with a correlation coefficient *r* > 0.8 in intergroups and intragroups were excluded.

(II) Then, model construction: the final retained features were included in the logistic regression analysis using a forward selection method, and image feature models of fat, calcification, and solid components were constructed for both inter-group and intra-group analyses.
(III) Lastly, model evaluation. The fit of models was assessed by Hosmer-Lemeshow test. The diagnostic performance of models was evaluated by area under the receiver operating characteristics curve (AUC).

**Fig. 2:**
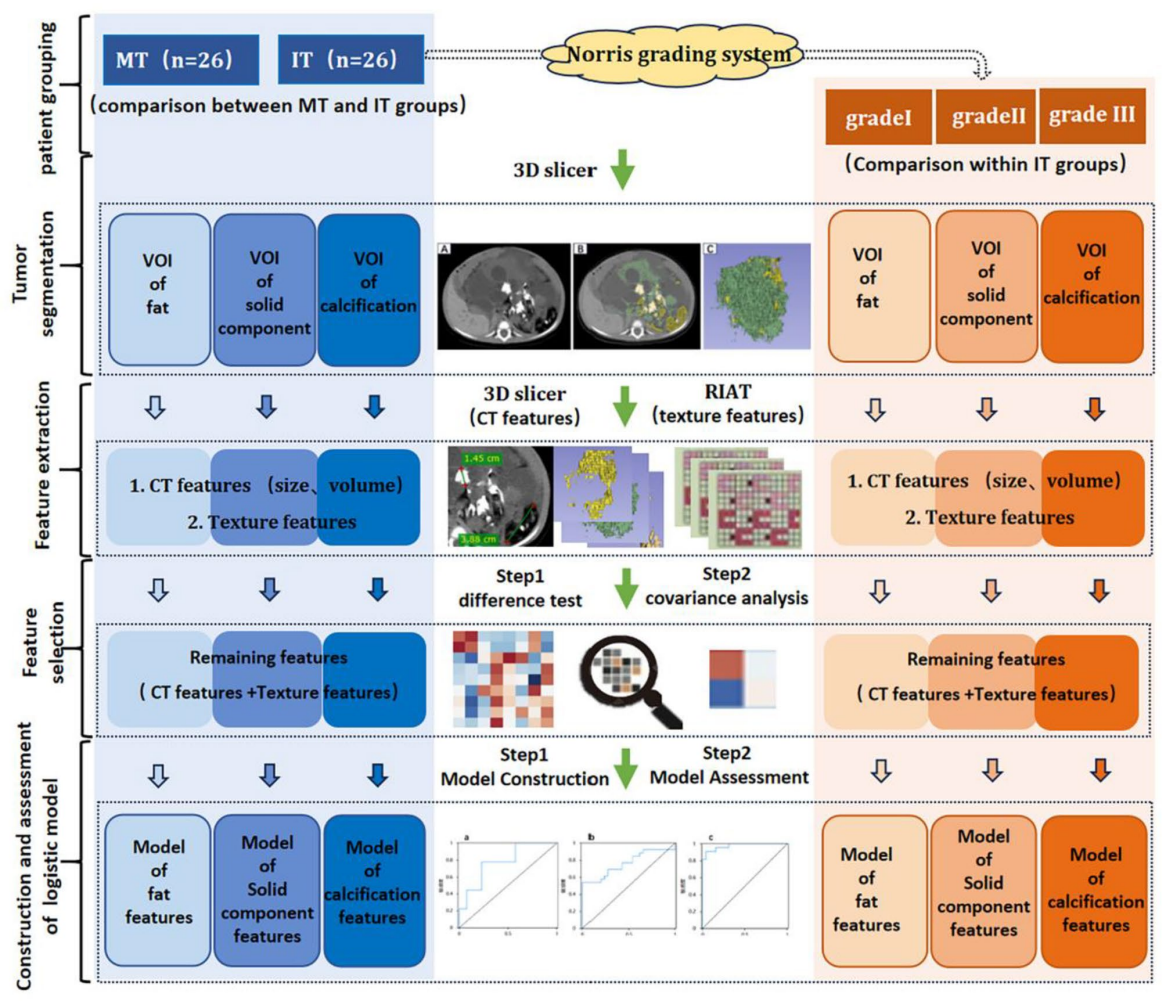
The flow chart of this study

### Statistical analysis

Categorical variables like sex were presented as percentages and analyzed via χ^2^ test. Numeric variables were presented as medians and quartiles and analyzed via the Mann-Whitney test for MT and IT intragroups and the Kruskal-Wallis test for grades I-III intergroups in IT. Difference tests, feature selection, model construction, and model evaluation are all carried out using SPSSAU (https://www.spssau.com.) [13]. If *p* < 0.05, differences are considered statistically significant.

## Results

### Patient data: (Table 1)

**Table 1:** Clinical characteristics of teratoma patients

Clinical characteristics		Teratoma		*p*	Immature teratoma			*p*
		MT	IT		Grade I	Grade II	Grade III	
Gender	male	7(26.92)	5(19.23)	0.51	1(14.29)	0(0.00)	4(26.67)	0.45
	female	19(73.08)	21(80.77)		6(85.71)	4(100.00)	11(73.33)	
Age/months		26.000(7.5,64.0)	60.000(3.0,120.0)	0.843	96.000(4.0,132.0)	67.500(3.0,132.0)	60.000(2.0,96.0)	0.665

*p<0.05,**p<0.01

The research enrolled a total of 52 patients with teratomas. The primary sites included gonads in 24 cases, mediastinum in 9 cases, retroperitoneum in 10 cases, abdomen in 2 cases, sacrococcygeal region in 3 cases, intracranial in 2 cases, temporal region in 1 case, and stomach in 1 case. Among them, 26 cases were classified as IT and 26 cases as MT. There were no statistically significant differences in gender and age between the IT and MT groups (*p* > 0.05). Within the IT group, the pathological grading of tumors was as follows: 7 cases of grade I, 4 cases of grade II, and 15 cases of grade III. There were no statistically significant differences in gender and age among the I-III grades within the IT group (*p* > 0.05).

### Differences in CT features: (Table 2)

**Table 2:** CT features of teratoma patients

CT features	Teratoma		*p*	Immature teratoma			*p*	
	MT	IT		Grade I	Grade II	Grade III		
Fat size/mm	40.500(16.3,70.5)	28.000(16.0,35.0)	0.15	32.000(15.5,46.5)	25.000(16.5,45.5)	26.500(15.3,37.8)		0.916
Fat volume/mm³	12390.000(1169.1,131755.5)	7549.520(2137.9,20699.8)	0.692	12835.300(1689.2,118952.6)	13316.205(3086.6,36338.1)	6981.670(1222.1,20385.2)		0.715
Calcification size/mm	14.000(8.0,20.0)	23.500(16.0,32.5)	0.012*	21.500(11.5,27.0)	36.500(19.8,43.5)	23.000(15.0,31.0)		0.254
Calcification volume/mm³	320.673(118.6,902.2)	2834.125(298.2,7954.2)	0.003**	5204.230(162.4,10704.5)	5775.730(1678.0,46462.3)	1603.401(256.0,7794.5)		0.454
Solid component volume/mm³	3664.490(627.3,18951.9)	71606.450(17499.9,122984.8)	0.000**	18597.300(14367.1,98812.9)	108561.800(35900.3,397467.5)	73859.300(17874.1,120447.0)		0.361

* *p*<0.05 ** *p*<0.01

Compared to the MT group, the IT group exhibited a larger calcification size (*p* = 0.012), higher calcification volume (*p* = 0.003), and higher solid component volume (*p* < 0.001). There were no inter-group differences in fat size and volume (*p* > 0.05). Within the I-III grades IT groups, there were no CT characteristics of fat, calcification, and solid components showing statistically significant differences (*p* > 0.05).

### Differences in texture features: (Table 3)

**Table 3:** NGTDM texture features of teratoma patients

Texture features	Teratoma		*p*	Immature teratoma			*p*
	MT	IT		Grade I	Grade II	Grade III	
NGTDM_coarseness_fat	0.001(0.0,0.0)	0.001(0.0,0.0)	0.427	0.001(0.0,0.1)	0.001(0.0,0.0)	0.001(0.0,0.0)	0.715
NGTDM_complexity_fat	27.980(8.4,115.9)	25.985(6.3,96.4)	0.667	96.389(46.7,119.3)	46.037(6.9,145.4)	17.094(4.7,46.8)	0.107
NGTDM_strength_fat	0.061(0.0,0.2)	0.048(0.0,0.2)	0.921	0.047(0.0,12.5)	0.066(0.0,0.4)	0.076(0.0,0.2)	0.92
NGTDM_contrast_fat	0.014(0.0,0.0)	0.008(0.0,0.0)	0.371	0.005(0.0,0.1)	0.009(0.0,0.0)	0.010(0.0,0.0)	0.618
NGTDM_busyness_fat	14.789(4.9,30.4)	17.527(5.6,29.1)	0.843	21.464(0.3,37.1)	11.749(5.1,18.6)	20.205(5.9,35.4)	0.655
NGTDM_coarseness_calcification	0.022(0.0,0.1)	0.003(0.0,0.0)	0.004**	0.002(0.0,0.0)	0.001(0.0,0.0)	0.007(0.0,0.0)	0.434
NGTDM_complexity_calcification	351.048(30.5,1144.1)	261.181(45.8,1084.4)	0.961	1086.437(90.8,1759.0)	428.959(131.6,769.3)	165.744(35.3,655.9)	0.361
NGTDM_strength_calcification	3.575(1.2,7.3)	0.897(0.4,1.4)	0.002**	1.042(0.8,2.9)	0.441(0.2,0.6)	0.897(0.4,1.5)	0.092
NGTDM_contrast_calcification	0.133(0.1,0.2)	0.055(0.0,0.1)	0.000**	0.057(0.1,0.1)	0.046(0.0,0.1)	0.056(0.0,0.1)	0.495
NGTDM_busyness_calcification	0.801(0.2,1.7)	2.070(0.9,4.1)	0.004**	1.615(0.6,3.0)	3.435(2.1,16.4)	1.766(1.0,4.3)	0.263
NGTDM_coarseness_solid	0.003(0.0,0.0)	0.000(0.0,0.0)	0.000**	0.001(0.0,0.0)	0.000(0.0,0.0)	0.000(0.0,0.0)	0.283
NGTDM_complexity_solid	12.121(0.2,43.0)	10.923(1.7,26.0)	0.901	26.843(4.7,34.3)	12.491(5.8,53.0)	7.186(1.2,23.1)	0.285
NGTDM_strength_solid	0.210(0.0,0.7)	0.046(0.0,0.1)	0.010**	0.121(0.1,0.2)	0.031(0.0,0.0)	0.042(0.0,0.1)	0.053
NGTDM_contrast_solid	0.002(0.0,0.0)	0.000(0.0,0.0)	0.000**	0.000(0.0,0.0)	0.001(0.0,0.0)	0.000(0.0,0.0)	0.656
NGTDM_busyness_solid	5.930(1.7,14.0)	26.556(14.9,56.2)	0.000**	15.281(4.1,25.3)	55.003(37.0,104.6)	27.857(15.8,61.5)	0.020*

* *p*<0.05 ** *p*<0.01

#### Texture features differences between IT and MT group

There were 22, 30, and 43 texture feature differences in fat, calcification, and solid components, respectively (*p* < 0.05). Previous studies have confirmed the value of NGTDM characteristics in distinguishing IT from MT [14]. Therefore, this study focused on analyzing the differences in NGTDM texture features. The results specifically showed that the busyness value of calcification and solid components was higher in IT group, while the coarseness, strength, and contrast values were lower in IT group (*p* < 0.005), with no variance in complexity among the groups. There were no differences in NGTDM texture features in fat between the MT and IT groups (*p* > 0.05).

#### Texture features differences within I-III grades IT group

There were no GLCM, GLDM, GLRLM, and GLZLM texture features differences in fat, calcification, and solid components within the IT groups (*p* > 0.05). Only the NGTDM_busyness feature of the solid component showed differences among ITs of different grades (*p* = 0.002), with grade II’s NGTDM_busyness value surpassing that of grade I.

### Construction and evaluation of three imaging feature models: (Table 4; Fig. 3)

**Table 4:** Comparison of three components models for identifying IT

Model	*p*	AUC(95%CI)	sensitivity	specificity
fat	0.014*	0.778(0.608 ~ 0.948)	0.778	0.769
calcification	0.001**	0.774(0.645 ~ 0.902)	0.538	1
solid component	0.000**	0.976(0.940 ~ 1.011)	0.909	0.962

* *p*<0.05 ** *p*<0.01

#### Fat radiology features model

There were 22 radiomic features with inter-group differences. 6 features were included in the logistic regression analysis finally. Results showed that GLRLM_GrayLevelNonUniformityNormalize was an independent risk factor for diagnosing IT (*p* < 0.05). The model had an ROC-AUC of 0.778 (95% CI: 0.645 to 0.902), a sensitivity of 0.778, a specificity of 0.769, and a Hosmer-Lemeshow test with *p* = 0.076 (*p* > 0.05), confirming a good fit.

#### Calcification radiology features model

There were 32 imaging features with inter-group differences, and 17 imaging features were finally included in the logistic regression analysis. Results showed that the total volume of calcification was an independent risk factor for diagnosing IT (*p* < 0.05). The model had a ROC-AUC of 0.774 (95% CI: 0.645 to 0.902), a sensitivity of 0.538, a specificity of 1, and a Hosmer-Lemeshow test with *p* = 0.152 (*p* > 0.05), confirming a good fit.

#### Solid component radiology features model

There were 44 radiomic features with inter-group differences. 13 imaging features were finally included in the logistic regression analysis. Results showed that the total volume, GLDM_GrayLevelNonUniformity, and GLRLM_RunVariance were all independent risk factors for diagnosis of IT (*p* < 0.05). The total volume and GLDM_GrayLevelNonUniformity were positively correlated with IT, while GLRLM_RunVariance was negatively correlated with IT. The model had a ROC-AUC of 0.976 (95% CI: 0.645 ~ 0.902), a sensitivity of 0.909, a specificity of 0.962, and a Hosmer-Lemeshow test with *p* = 0.905 (*p* > 0.05), confirming a good model fit.

Based on the difference test results of IT I-III intragroup comparisons, a logistic regression was developed, identifying that the NGTDM_busyness feature was not an independent risk factor for diagnosing grade II(*p* > 0.05).

**Fig. 3:**
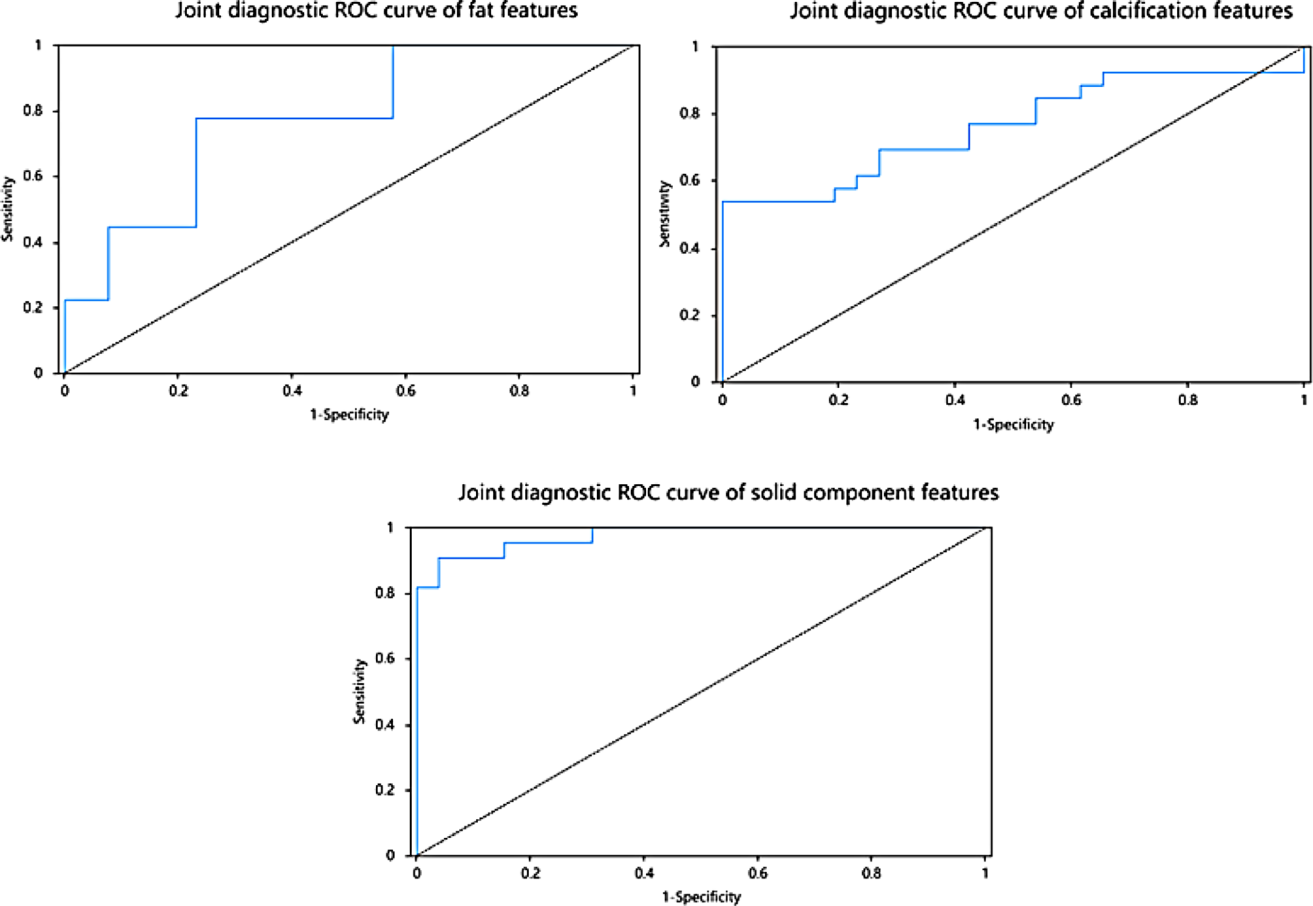
Joint diagnostic ROC curve of intratumoral components. fat features, calcification features, and solid component features.ROC Receiver Operating Characteristic

## Discussion

Immature teratoma (IT) is heterogeneous malignant tumor, which is prone to metastasis and recurrence [1]. Tumor grading is critical for predicting prognosis and guiding clinical management [15]. This study demonstrated the efficacy of computed tomography texture analysis (CTTA) in IT detection and grading, particularly highlighting the value of neighborhood gray tone difference matrix (NGTDM) texture features. Among tumor components, solid components showed the greatest diagnostic importance.

Teratomas, derived from pluripotent stem cells, include tissues from the three germ layers and typically present as fat, calcification, and solid components in imaging [16]. IT contains more incompletely differentiated tissues. With higher grades, IT contains a greater amount of immature tissues, indicating a more complex degree of differentiation. Therefore, higher-grade IT exhibits greater heterogeneity compared to lower-grade IT. CTTA can accurately capture the microscopic heterogeneity differences [7], making it applicable for IT diagnosis and grading.

Our findings align with previous research by Nakamori et al. [7], who used CTTA to analyze calcification and fat distribution in teratomas, confirming the heterogeneity of calcifications in IT. In this study, the results of NGLCM features were highly consistent with their research findings, both demonstrating higher busyness in IT compared to MT, while coarseness and contrast were lower in IT. Furthermore, our study analyzed the texture differences in solid components, yielding similar NGTDM feature results. As the NGTDM features quantify the overall difference in average gray levels between voxels and their neighboring voxels, they reflect the spatial heterogeneity of different grayscale levels within the lesion [17]. Combining the findings of previous study, it can be concluded that both calcification and solid components in IT exhibit significant heterogeneity, and NGTDM features demonstrate high stability, making them useful for preoperative diagnosis of IT.

Accurate grading of IT is essential for predicting patient prognosis. A large-scale study of 1307 ovarian IT cases confirmed that tumor grading is a key predictor of prognosis, with survival rates of 98.7%, 95.8%, and 91% for grades I, II, and III, respectively [18]. However, previous research has identified few positive indicators for IT grading. Norris et al. [9] reported tumor size did not correlate with the tumor grade.Shinkai et al. [15] found that patients with high-grade IT tend to be older and have higher AFP levels than those with low-grade IT, but no statistical analysis was performed. Yamaoka et al. [19] examined the relationship between the amount of solid components and IT grading, revealing an insignificant correlation (*r* = 0.266). Similarly, in this study, the size and total volume of fat, calcification, and solid components, are not statistically significant for grading predictions within the IT groups. It’s speculated that there are limited CT features visible to the naked eye. However, CTTA revealed that the NGTDM_busyness feature of the solid component not only differed between the IT and MT groups but also showed higher values in grade II compared to grade I within the IT group (*p* = 0.020). This highlights the value of the NGTDM_busyness feature in the preoperative identification and grading of IT.

This study also demonstrated that calcification and solid component content can distinguish IT from MT, with IT exhibiting higher levels of these components (*p* < 0.05), while fat content showed no significant difference. MT typically presents as cystic lesions with spherical fat and minimal calcifications, while IT primarily manifests as solid lesions with scattered fat and calcified particles [20, 21]. The insufficient tissue differentiation in IT may drive increased calcification and solid component formation. In contrast, fat in IT appears to have reached a relatively mature differentiation stage [22], which may explain the lack of significant differences in fat content between MT and IT. However, our results differ from those of Nakamori et al., who manually counted calcifications and fat and found both to be more prevalent in IT [14]. This discrepancy may be due to the difficulty in identifying small calcifications and fat in IT. In our study, the use of 3D Slicer software enabled more precise quantitative assessment of intratumoral components. Based on logistic regression analysis, the total content of calcifications and solid components were identified as independent diagnostic risk factors for IT. Notably, solid components achieved an ROC-AUC of 0.976 (95% CI: 0.645 ~ 0.902), consistent with previous research. Zhou et al. constructed a logistic regression model identifying intratumoral solid components ≥ 50%, polymorphic calcifications, and scattered adipose tissue as independent risk factors for IT, with solid components ≥ 50% as the most significant [4]. By objectively quantitatively analyzing intratumoral features using 3D Slicer, this study further confirms the crucial diagnostic value of solid components in differentiating IT from MT.

However, several limitations exist in this study. The relatively low incidence of IT and single-center design limited our sample size and may have introduced potential selection bias. Future studies should expand the sample and include external validation. The analysis didn’t fully consider textural differences at various tumor sites. Also, to ensure reproducibility, only 75 stable original texture features were analyzed, not covering all known ones. Future research should delve deeper into this area and accumulate more experience to further verify and explain the relevance and mechanisms of textural features in clinical practice. Furthermore, our study mainly focused on preoperative imaging features and their correlation with tumor grading, without incorporating clinical variables like patient age, tumor size, or serum tumor markers like AFP. Future studies could combine multi - dimensional data to create more comprehensive predictive models for IT grading and prognosis.

## Conclusions

In summary, This study addresses the critical gap in preoperative identification and grading of IT by demonstrating the efficacy of CTTA, particularly NGTDM texture features, in characterizing tumor heterogeneity. Solid components emerged as the most robust diagnostic indicator, aligning with the pathological basis of IT. In clinical practice, CTTA could serve as a valuable adjunct to conventional imaging, enabling earlier and more accurate grading of IT, particularly in resource-limited settings where histopathological expertise is scarce. For future research, these results pave the way for the development of personalized treatment algorithms, potentially reducing overtreatment in low-risk cases and improving outcomes in high-risk patients. Additionally, exploring the application of CTTA in other pediatric tumors with heterogeneous histology (e.g., neuroblastoma or Wilms tumor) could broaden the translational potential of this technique. Notably, NGTDM_busyness in solid components not only differentiated IT from MT but also increased with higher IT grades. These unexpected findings suggest that this textural biomarker sensitively captures subtle histological changes and predicts tumor behavior beyond traditional morphological assessments.

## Data Availability

No datasets were generated or analysed during the current study.

## Abbreviations

CT: Computed tomography
CTTA: Computed tomography texture analysis
MT: Mature teratomas
IT: Immature teratomas
ROC: Receiver operating characteristic
AUC: Area under the curve
NGLCM: Neighborhood gray tone difference
